# Specificity of ε and Non-ε Isoforms of *Arabidopsis* 14-3-3 Proteins Towards the H^+^-ATPase and Other Targets

**DOI:** 10.1371/journal.pone.0090764

**Published:** 2014-03-06

**Authors:** Roberta Pallucca, Sabina Visconti, Lorenzo Camoni, Giovanni Cesareni, Sonia Melino, Simona Panni, Paola Torreri, Patrizia Aducci

**Affiliations:** 1 Department of Biology, University of Rome “Tor Vergata”, Rome, Italy; 2 Department of Sciences and Chemical Technologies, University of Rome “Tor Vergata”, Rome, Italy; 3 Department DiBEST, University of Calabria, Rende, Italy; 4 Department of Cell Biology and Neuroscience, Istituto Superiore di Sanità, Rome, Italy; 5 IRCSS, Research Institute “Fondazione Santa Lucia”, Rome, Italy; University of South Florida College of Medicine, United States of America

## Abstract

14-3-3 proteins are a family of ubiquitous dimeric proteins that modulate many cellular functions in all eukaryotes by interacting with target proteins. 14-3-3s exist as a number of isoforms that in *Arabidopsis* identifies two major groups named ε and non-ε. Although isoform specificity has been demonstrated in many systems, the molecular basis for the selection of specific sequence contexts has not been fully clarified. In this study we have investigated isoform specificity by measuring the ability of different *Arabidopsis* 14-3-3 isoforms to activate the H^+^-ATPase. We observed that GF14 isoforms of the non-ε group were more effective than ε group isoforms in the interaction with the H^+^-ATPase and in the stimulation of its activity. Kinetic and thermodynamic parameters of the binding of GF14ε and GF14ω isoforms, representative of ε and non-ε groups respectively, with the H^+^-ATPase, have been determined by Surface Plasmon Resonance analysis demonstrating that the higher affinity of GF14ω is mainly due to slower dissociation. The role of the C-terminal region and of a Gly residue located in the loop 8 and conserved in all non-ε isoforms has also been studied by deletion and site-specific mutagenesis. The C-terminal domains, despite their high divergence, play an auto-inhibitory role in both isoforms and they, in addition to a specific residue located in the loop 8, contribute to isoform specificity. To investigate the generality of these findings, we have used the SPOT-synthesis technology to array a number of phosphopeptides matching known or predicted 14-3-3 binding sites present in a number of clients. The results of this approach confirmed isoform specificity in the recognition of several target peptides, suggesting that the isoform specificity may have an impact on the modulation of a variety of additional protein activities, as suggested by probing of a phosphopeptide array with members of the two 14-3-3 groups.

## Introduction

14-3-3 proteins are a family of evolutionary conserved dimeric proteins that accomplish a wide range of regulatory roles in eukaryotes, including cell cycle control, mitogenesis, and apoptosis [1]. In plants, these proteins regulate primary metabolism, ion transport, cellular trafficking, gene transcription and hormone signalling [2], [3].

14-3-3 proteins exist as multiple isoforms and the common theme in their mechanism of action is the ability to associate with target proteins, through binding to consensus motifs [4], [5]. Generally, 14-3-3 binding to the target occurs at phospho-Ser- or phospho-Thr-containing motifs, RSX(pS/pT)XP and RXY/FX(pS/pT)XP, defined as mode I and mode II motifs, respectively [5]. More recently, a mode III C-terminal binding motif, where the residue preceding the carboxy-terminal end is a phosphorylated Ser or Thr, has also been defined [6]. A large number of 14-3-3 target proteins have thus far been identified, both in animals and in plants [7]. For some of them the 14-3-3 binding site has also been determined [8].

The crystal structures solved for human [9]–[11] and tobacco [12] 14-3-3 isoforms demonstrated that 14-3-3 proteins share a very similar tridimensional architecture, with a highly conserved amphipathic groove involved in the ligand binding.

In *Arabidopsis thaliana* thirteen 14-3-3 isoforms are expressed [13] and are often referred to as GF14 proteins, since they were initially identified as a part of a G-box binding complex [14]. Comparison of the *Arabidopsis* 14-3-3 isoforms reveals a high degree of amino acid identity, being the differences confined at the N and C termini [15]. Based on a phylogenetic analysis, *Arabidopsis* 14-3-3s can be divided into two major groups named ε and non-ε. The 14-3-3 ε group has five members – μ (mu), ε (epsilon), π (pi), ι (iota), and ο (omicron) – while the non-ε group has eight members – κ (kappa), λ (lambda), ψ (psi), ν (nu), υ (upsilon), ω (omega), φ (phi), and χ (chi) –. The relatively large number of 14-3-3 isoforms, as well as the abundance of 14-3-3 target proteins, has raised the issue of functional specificity. It is not clear whether 14-3-3s can accomplish specific functions by binding their targets in an isoform-specific manner. Structural analysis does not provide support for the hypothesis of isoform specificity, since the solvent exposed surface of the target-binding pocket is highly conserved among isoforms [11], [12], [16], [17]. In several systems 14-3-3 isoforms were shown to be experimentally interchangeable [18], thus suggesting functional redundancy. In this respect, the isoform specificity demonstrated in *in vivo* studies may be the result of differences in the expression patterns rather than residing in the different biochemical properties of 14-3-3 proteins. In accordance, differential expression of 14-3-3 isoforms was observed in different tissues and organs [19], as well as during plant development or in response to different environmental stimuli [20].

On the other hand, several pieces of evidence suggest that 14-3-3 isoforms specifically interact with different target proteins. To mention some examples, in plants, the nitrate reductase [21], [22], the plasma membrane H^+^-ATPase [23], [24], the sucrose-phosphate synthase [25], phototropin 1 [26] and, more recently, the ABA-responsive-element Binding Factor (ABF) [27]. Interestingly, the differential subcellular distribution of 14-3-3s seems to be dependent upon specific interactions with cellular clients [28].

The reported functional specificity somewhat contrasts with the observation that the target binding pocket is highly conserved in the different isoforms. It has been proposed that the C-terminal region of 14-3-3s, characterized by a high level of divergence among isoforms, can play a role in client binding [29–32). In addition, 14-3-3s binding to several targets requires the presence of divalent cations and polyamines [33], [34] that seem to modulate phosphopeptide binding by affecting the conformation of an EF hand-like motif within loop 8 [35] of the 14-3-3 structure. Accordingly, mutation of a specific amino acid residue in loop 8 affects the divalent cation binding and alters client binding selectivity [21], [36].

In the present work, we studied the molecular mechanisms underlying the *Arabidopsis* 14-3-3 isoforms recognition specificity in the modulation of the activity of the plasma membrane H^+^-ATPase, one of the best characterized plant 14-3-3 client proteins [37]. In addition, we used the SPOT-synthesis technology to assemble a peptide array representing different known or putative 14-3-3 binding sites. Results demonstrate that H^+^-ATPase preferentially interacts with non-ε 14-3-3 isoforms and show the role of the C-terminal region in the isoform specificity. Furthermore, peptide array studies identified additional targets that are bound by 14-3-3s in an isoform-specific manner.

## Materials and Methods

### Chemicals

[γ-^32^P]ATP (specific activity 110 TBq mmol^−1^) was obtained from Amersham Biosciences (Uppsala, Sweden).

The bL15Vp peptide biotinyl-LKGLDIDTIQQNYTpV (Tp, phosphothreonine) was synthesized by Neosystem (Strasbourg, France).

Streptavidin-agarose magnetic beads, glutathione-sepharose resin, the catalytic subunit of protein kinase A and thrombin were obtained from Sigma (St. Louis, MO, USA).

Restriction enzymes were obtained from Roche Diagnostics (Mannheim, Germany). Pfu turbo DNA polymerase was obtained from Stratagene (La Jolla, CA, USA).

Chemicals for SDS–PAGE were obtained from Bio-Rad (Hercules, CA, USA).

### Plant material

Maize caryopses (*Zea mays* L. Hybrid PR35P12) from Pioneer Italia Hi Bred (Parma, Italy) were germinated and seedlings were grown in the dark for five days, as previously described [38].

### Purification of plasma membrane from maize roots

Two-phase partitioned plasma membranes were obtained from 20 g of maize roots as previously described [38].

### Purification of endoplasmic reticulum from yeast expressing AHA1

The *Arabidopsis* H ^+^- ATPase isoform 1 (AHA1) was expressed in *Saccharomyces cerevisiae*, as previously described [39]. Following cell homogenization, membranes were purified by differential centrifugation and endoplasmic reticulum (ER), containing most of the AHA1, was isolated by sucrose gradient centrifugation [38].

### Isolation of GF14 isoform cDNA

*Arabidopsis* GF14ε (At1g22300), ω(At1g78300), χ(At4g09000), κ(At5g65430), λ(At5g10450), μ (At2g42590) and ο (At1g34760) cDNAs were obtained by RT-PCR. Total RNA isolated from *Arabidopsis* seedlings using the RNeasy Plant Mini RNA kit (Qiagen) was subjected to retro-transcription by using the SuperScript First-strand Synthesis system (Invitrogen) and PCR with primers corresponding to the coding region sequences, with added terminal EcoRI and BamHI restriction sites for subsequent cloning. All amplified cDNAs were controlled by DNA sequencing (Eurofins MWG Operon, Ebersberg, Germany).

### Site-directed mutagenesis of GF14 proteins

pGEX-2TK carrying the GF14ε and the GF14ω cDNA were used as templates for mutagenesis of GF14ε and GF14ω proteins. Site-directed mutagenesis of GF14ε and GF14ω proteins was performed by using the ‘QuickChange site-directed mutagenesis method’ (Stratagene) following the manufacturer's protocol.

The GF14εΔC and GF14ωΔC mutants, deleted of the last 20 and 23 amino acids, were obtained by changing the Asp235 codon of GF14ε and the Asp237 codon of GF14ω, into the Stop codons, thereby preventing translation of the last 20 and 23 amino acids respectively.

The GF14εN211G and the GF14ωG213N mutants were obtained by changing the Asn211 codon of GF14ε into the Gly codon and the Gly213 codon of GF14ω into the Asn codon, respectively.

The mutagenic primers used were (the mutation generated is underlined): 5′ CTCACCTTGTGGACTTCATAGCTTAATGAGGAAGGAGATG 3′ and its reverse complement for GF14εΔC and 5′ CAATCTCACTCTCTGGACATCTTAGATGCAGGATGATGCTG 3′ and its reverse complement for GF14ωΔC; 5′ GCTGAACTTGACAGCCTCGGCGAGGAATCATACAAAGACAGC 3′ and its reverse complement for GF14εN211G and 5′ GCAGAGTTGGACACTCTTAATGAAGAGTCATACAAAGACAG 3′ and its reverse complement for GF14ωG213N.

After 18 cycles of PCR (30 s at 95°C, one minute at 55°C, seven minutes at 68°C), 10U of DpnI were added to the mixtures to digest the GF14ε and the GF14ω cDNA templates and reactions were carried out at 37°C for 2 h. A 20 µl aliquot of each mixture was used to transform *E. coli* DH5a competent cells. Incorporation of mutations was controlled by DNA sequencing.

### Expression of recombinant proteins in *E. coli*

Wild-type and mutated GF14 proteins were expressed in *E. coli* BL21(DE3) strain as Glutathione S-Transferase (GST)-fusion proteins using the pGEX-2TK vector, while the C-terminal domain of the Maize H ^+^-ATPase isoform 2 (MHA2) (corresponding to the last 103 amino acids) was expressed as a GST-fusion protein using pGEX-2T following the procedure described by Fullone et al. [40].

### ^32^P-labeling of GST fusion proteins

Wild type and mutated GF14 proteins were labelled with [γ^32^P]-ATP on the phosphorylation site present at the junction between GST and the cloned protein, using the catalytic subunit of PKA as already described [40]. Specific activities of ^32^P-labeled proteins were similar (about 90 GBq mmol^–1^).

### Overlay assay

The overlay assay was carried out according to Fullone et al. [40] with minor modifications; 3 µg GST-fused C-terminal domain of H^+^-ATPase or 20 µg of maize plasma membranes, containing the H^+^-ATPase, were separated on SDS-PAGE and blotted on nitrocellulose membrane by semidry electroblotting (Bio-Rad). The membrane was blocked with 5% fatty acid-free milk in 25 mM HEPES-OH, 75 mM KCl, 5 mM MgCl_2_, 1 mM dithiothreitol, 0.1 mM EDTA, 0.05% Tween-20, pH 7.5 (buffer HT) and then cut into identical strips which were incubated overnight at 4°C in the same buffer containing 3% fatty acid-free milk, 0.1 μM ^32^P-labeled GF14 isoforms (corresponding to 9 kBqml^–1^) and, where indicated, 10 μM fusicoccin (FC). The nitrocellulose membrane was then extensively washed three times with buffer HT and radioactivity detected by autoradiography. Densitometric analysis was performed using the ImageJ image-processing program [41] Densitometric data are expressed as a percentage of the maximum Integrated Densitometric Value (the product of area and mean gray value).

### Binding to bL15Vp peptide

The bL15Vp peptide (0.05 nmol) was immobilized onto 20 µl of streptavidin-agarose magnetic beads and incubated in 50 µl of buffer HT (containing or without Mg^2+^) with 0.1 nmol of ^32^P-labeled GF14 isoforms or GF14 mutants (1.4 kBq μg^–1^) for 60 min at room temperature in the absence or in the presence of 10 µM FC. The resin was then centrifuged at 2000 g for five minutes and washed three times with 1 ml of buffer HT. Resin bound radioactivity was measured in a liquid scintillation β-counter (Tri Carb 2100TR Packard).

### Surface Plasmon Resonance

The kinetic parameters, association rate constants (k_on_) and dissociation rate constants (k_off_), were determined using the BIAcoreX system for Surface Plasmon Resonance (SPR) detection.

Biotinylated peptide bL15Vp (600 resonance units) was captured on an Biacore SA sensor chip precoated with streptavidin. The analyte (GF14ε or GF14ω) was applied in the concentration ranges of 10 µM–7.8 nM. The interaction was followed in real time. Experiments were performed in HT buffer in the absence or presence of 10 µM FC at 25°C. Solutions of GF14 protein in the running buffer were injected into the flow cell and passed over the peptide surfaces at a continuous flow rate of 30 µl/min for two minutes. Dissociation was monitored for 5 min. The sensor surface was regenerated for the next sample using a 2 µl pulse of 50 mM glycine-HCl pH 2 to remove GF14 protein bound to the immobilized peptide.

All sensorgrams were corrected for bulk refractive indexes by reference – subtracting with sensorgrams from a flow cell in which only streptavidin was immobilized. The data were evaluated using BIAevaluation 3.1 (Biacore AB). To obtain K_D_ and Rmax values, a 1∶1 Langmuir binding model was fitted to the sensorgrams.

### Phosphohydrolytic activity

The AHA1 phosphohydrolytic activity of yeast ER membranes was assayed according to Camoni et al. [42] with minor modifications: 10 μg of sucrose gradient-purified yeast ER were pre-incubated with different concentrations of wild-type or mutated GST-GF14 isoforms (ranging from 0 to 4 μM) in 500 μl of 50 mM Tris-MES, 5 mM MgCl_2_, 50 mM KNO_3_, 5 mM NaN_3_, 0.2 mM ammonium molybdate, pH 7.2 (buffer A) in the presence of 10 μM FC. After a 20 minutes incubation, 2 mM ATP was added.

### Tryptophan fluorescence spectroscopy

Tryptophan fluorescence spectra were recorded with a Perkin Elmer luminescence spectrometer LS50B at 25°C using 1.5 µM GF14 in 1 ml of 25 mM Tris-HCl, 150 mM NaCl, pH 7.5. The excitation wavelength was 280 nm and the emission wavelength ranged from 300 to 500 nm.

### Circular dichroism analysis and protein secondary structure prediction

Circular Dichroism (CD) measurements were performed using a Jasco 600 spectropolarimeter, equipped with a thermal controller calibrated with camphor-sulfonic acid. Far-UV CD experiments were carried out to explore the conformation of the GF14 proteins (5 μM). CD spectra were obtained between 200 and 250 nm using a path-length of 0.1 cm with a time constant of 1.0 s, a 2 nm bandwidth and a scan rate of 2 nm min^−1^ and at 10 mdeg sensitivity. Each spectrum was averaged over four scans and subjected to smoothing following subtraction of the buffer background. The measured ellipticity data were converted to mean molar ellipticity ([*θ*], degcm^2^ dmol^−1^).

Estimation of the secondary structure contents was done with the GOR secondary structure prediction method version IV [43].

### Peptide array

The peptides, 13 aminoacids long, were synthesized according to standard solid phase synthesis protocols [44], using an automatic SPOT synthesizer (Intavis, Koeln, Germany). In this approach, peptides are synthesized in array format, bound to cellulose membranes, which are activated with amino PEG (Intavis). The chemistry uses Fmoc protected amino-acids, with protected side chains, which are deprotected at the end of the synthesis.

Before use, the membranes were wet in ethanol and washed repeatedly in PBS. Membranes were blocked overnight at 4°C in buffer HT containing 5% BSA (blocking buffer).

The binding assay was performed incubating the membranes with 0.2 µM GST-fused GF14 isoforms, or GST alone as a negative control, in blocking buffer overnight at 4°C. After washing membranes three times for 10 minutes with HT buffer, the anti-GST peroxidase-conjugated antibody was added 1/1000 in blocking buffer and incubated for 2 h. Membranes were washed three times with HT buffer, and quantification of peptide bound GF14 proteins was carried out using a chemo-luminescence substrate and the LAS-3000 instrument (Luminescent Image Analyzer, Fujifilm).

Densitometric analysis of positive spots was performed using the ImageJ image processing program [41] and data expressed as Integrated Densitometric Value (the product of the area and mean gray value).

### Analytical methods

Protein concentration was determined by the method of Bradford [45], using bovine serum albumin as the standard.

### Statistics

Data were analyzed using Microsoft Excel and assessed for significance by the Student's *t*-test.

## Results

### Non-ε GF14 isoforms preferentially interact with the H^+^-ATPase

Some isoforms belonging to the ε and non-ε groups were used to investigate their ability for binding with and activating the H^+^-ATPase. GF14χ, GF14κ, GF14λ and GF14ω isoforms, belonging to the non-ε group, and GF14ε, GF14 μ and GF14ο isoforms, belonging to the ε group were expressed in *E. coli* as GST-fusion proteins, purified by affinity chromatography and used in overlay experiments with the H^+^-ATPase. The plasmalemma H^+^-ATPase was immobilized onto nitrocellulose membrane and incubated with identical amounts of ^32^P-labelled GF14 isoforms. The experiment was performed also in the presence of fusicoccin (FC), a fungal toxin known to activate the H^+^-ATPase by strongly stabilizing its interaction with 14-3-3 proteins [46]–[48].

**Figure 1:**
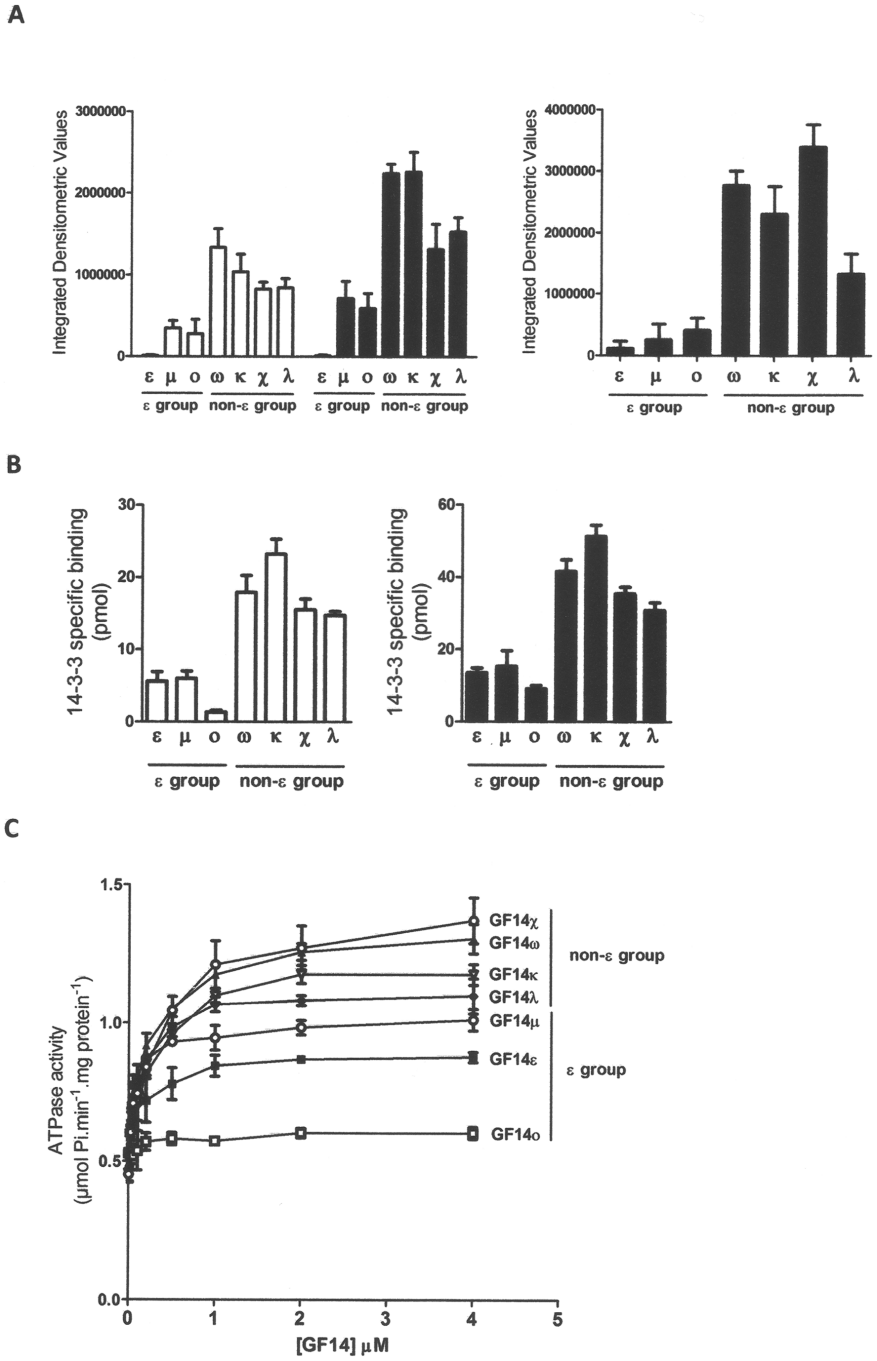
Comparison of different ε and non-ε isoforms for the ability to bind and activate the H^+^-ATPase. A. Densitometric analysis of a relative amount of GF14 isoforms bound to the H^+^-ATPase (left panel) and to its recombinant C-terminal domain (right panel) estimated from overlay assays. Ten µg of plasma membrane preparation from maize roots or 1 µg of the recombinant GST-fused MHA2 C-terminal domain were subjected to the SDS-PAGE, then blotted onto nitrocellulose membrane and incubated with 0.1 μM ^32^P-labeled-GF14 isoforms in the presence or in the absence of 10 µM FC. Densitometric analysis was performed on four independent overlay experiments and data are the means ± S.E. B. Peptide binding assay: 0.05 nmol of bL15Vp biotinyl-peptide reproducing the last 15 amino acids of MHA2 H^+^-ATPase, phosphorylated on the threonine residue at position −1 from the C-terminus, were immobilized onto streptavidin–agarose magnetic beads and incubated with 0.1 nmol ^32^P-labeled-GF14 isoforms in the presence or in the absence of 10 µM FC. After washing, the amount of peptide-associated 14-3-3 was estimated by measuring the beads bound radioactivity. Data are the means ± S.E. of three independent experiments. C. Comparison of GF14 isoforms ability to stimulate the H^+^-ATPase: phosphohydrolytic activity of H^+^-ATPase was determined by incubating 10 µg of ER yeast vesicles, containing AHA1, with GF14 isoforms at different concentrations (ranging from 0 to 4 μM) in the presence of 10 µM FC. Data are the means ± S.E. of four independent experiments.

Densitometric analysis of bands detected in the overlay experiments (see Figure 1A) shows that GF14ε, GF14 μ and GF14ο interacted with the H^+^-ATPase (left panel) at a very low extent, both in the presence and in the absence of FC. On the contrary, non-ε isoforms were all able to interact with the H^+^-ATPase in both conditions.

14-3-3 proteins can also interact with the H^+^-ATPase in a phosphorylation-independent manner, but exclusively in the presence of FC [40]. GF14 isoforms were compared in their interaction with the C-terminal domain (103 amino acids) of the H^+^-ATPase, expressed in *E. coli* as a GST-fusion protein and therefore not phosphorylated. A similar result was obtained, as shown in Figure 1A (right panel), non-ε isoforms bound to C-terminal domain, at difference with all ε group isoforms.

Overlay data were corroborated by results obtained with a phosphorylated biotinyl-peptide (bL15Vp) reproducing the last 15 residues of the MHA2 H^+^-ATPase. This peptide contains the YpTV 14-3-3 binding sequence and it has previously been reported to bind 14-3-3 proteins in pull down assay [32]. bL15Vp was immobilized onto streptavidin-agarose beads and then incubated with ^32^P-labelled GF14 isoforms in the presence and absence of FC. The results, reported in Figure 1B, showed that GF14 non-ε isoforms interacted with the peptide much more efficiently than ε isoforms and the difference between the two groups was significant both in the absence and in the presence of FC.

GF14 isoforms were also assayed for their ability to activate the H^+^-ATPase. A recombinant AHA1 H^+^-ATPase isoform was expressed in yeast where most of the functional enzyme is located at the endoplasmic reticulum [49] and can be significantly and reproducibly stimulated by exogenous 14-3-3, if FC is added [50]. AHA1 H^+^-ATPase was incubated with different amounts of GF14 isoforms, in the presence of 10 µM FC. The effect of different concentrations of GF14 isoforms on the phosphohydrolytic activity of recombinant AHA1 H^+^-ATPase is shown in Figure 1C. All the non-ε isoforms tested were more active than the ε isoforms. Among the ε isoforms, GF14 μ resulted more effective in stimulating the H^+^-ATPase, while GF14ε and GF14ο displayed a very low activity at higher concentrations as well, which is in accordance with interaction data reported above.

Results obtained strongly suggest that the H^+^-ATPase binding ability of GF14 isoforms depends on the phylogenetic group to which they belong [13].

Two GF14 isoforms, GF14ε and GF14ω, representative of the ε and non-ε groups, respectively, were selected to better characterize the interaction with the H^+^-ATPase.

Firstly, a kinetic and thermodynamic study of the interaction was performed by SPR analysis using the bL15Vp phosphopeptide. The kinetic and dissociation constants, determined for the interaction in the absence and in the presence of FC, are reported in Table 1. As shown, in the absence of FC the equilibrium dissociation constant of GF14ω was about 5 fold lower than for GF14ε. This different affinity is mainly due to a slower dissociation rate for GF14ω, being that the k_off_ was about 10 fold lower for the GF14ω interaction, while the k_on_ resulted of the same order of magnitude for both isoforms. In the presence of FC, the difference in the equilibrium dissociation constants was even more evident and the GF14ω affinity to bL15Vp peptide was increased more than 10 fold, in accordance with what had already been determined for maize GF14-6 isoform by SPR [46] and for the tobacco 14-3-3c isoform by isothermal titration calorimetry [12]. The FC effect was on the dissociation phase, being that the k_off_ was strongly reduced by the toxin and the k_on_ almost unaffected.

**Table 1:** Kinetic and thermodynamic parameters of the GF14ε and GF14ω interaction with the H^+^-ATPase.

GF14 isoform	k_on_ (M^−1^. s^−1^)	k_off_ (s^−1^)	K_D_ (nM)
GF14ε	5.63×10^4^	1.27×10^−2^	231
GF14ω	7.63×10^4^	3.82×10^−3^	53
GF14ε + FC	2.17×10^4^	3.07×10^−3^	124
GF14ω + FC	1.84×10^4^	5.43×10^−5^	4.1

The kinetic parameters, association rate constants (k_on_) and dissociation rate constants (k_off_), were determined using the BIAcoreX system for SPR detection. Biotinylated peptide bL15Vp (600 resonance units) was captured on an Biacore SA sensor chip precoated with streptavidin. GF14ε or GF14ω were applied in the concentration ranges of 10 µM–7.8 nM in the absence or presence of 10 µM FC at 25°C, with a flow rate of 30 µl/min. Dissociation was monitored for five minutes. The data were evaluated using BIAevaluation 3.1 (Biacore AB). To obtain K_D_ and Rmax values, a 1∶1 Langmuir binding model was fitted to the sensorgrams.

**Figure 2:**
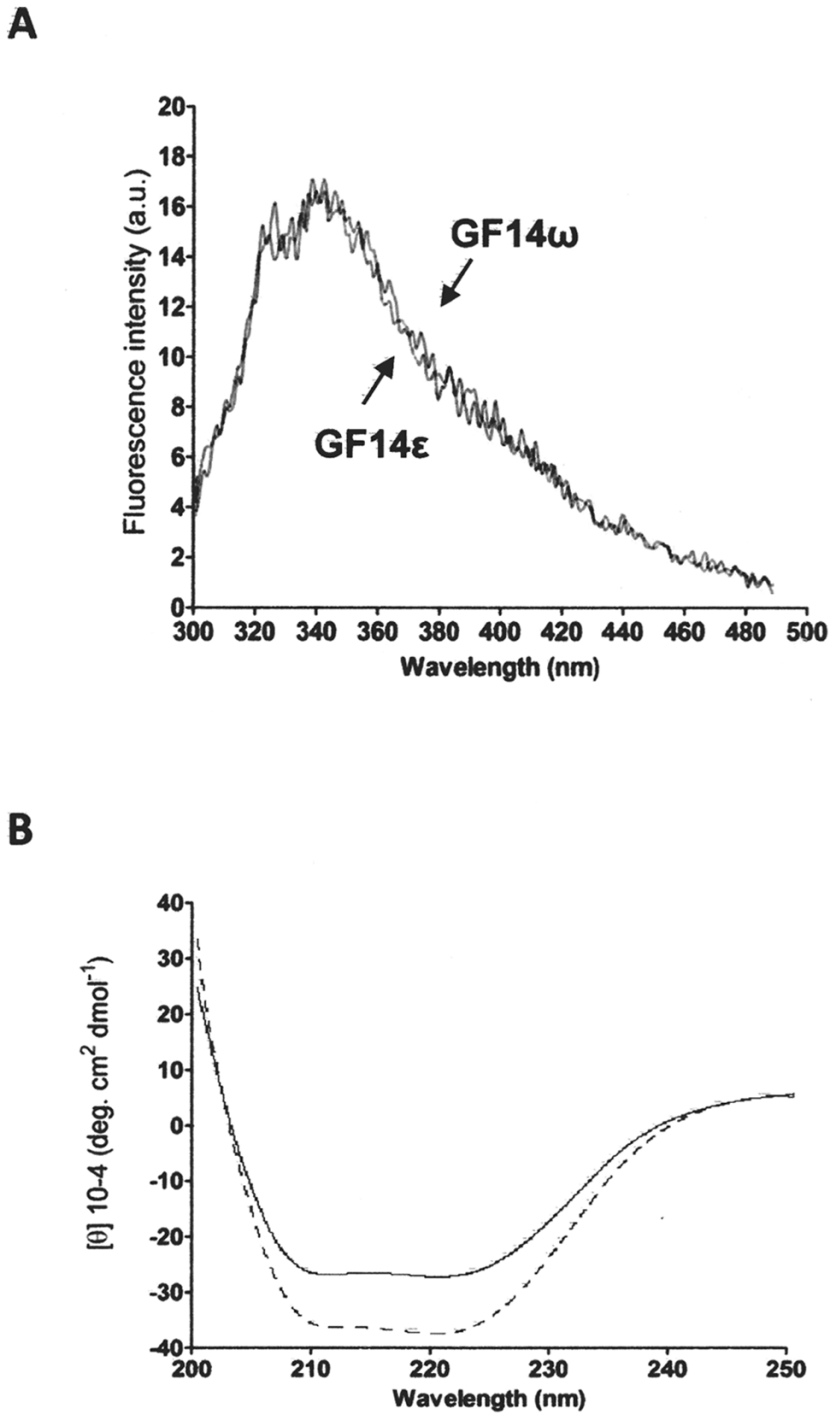
Tryptophan fluorescence emission spectra and circular dichroism spectra of GF14ω and GF14ε. A. Tryptophan fluorescence spectra were obtained at the excitation wavelength of 280 μM protein. B. Far-UV CD spectra of GF14ω and GF14ε were obtained in the 200 and 250 nm range using 5 µM protein. Each spectrum was averaged over four scans and subjected to smoothing following subtraction of the buffer background. The measured ellipticity data were converted to mean molar ellipticity ([*θ*], degcm^2^ dmol^−1^). Continuous line, GF14ε; dotted line, GF14ω.

A control on the possible role of protein folding in the different behaviour of the two isoforms was performed by comparing the tryptophan intrinsic fluorescence spectra of GF14ω and GF14ε. As shown in Figure 2A, the emission spectra of GF14ω and GF14ε were superimposed, in accordance with the number and position of all tryptophan residues conserved in the two GF14 isoforms, thus indicating that they shared a very similar folding. Their secondary structure was also analysed by circular dichroism spectroscopy. The Far-UV CD spectra (Figure 2B) shown that the α-helices were the major component in the secondary structure for both isoforms and were comparable to previously reported spectra [21], [33]. The α-helical content of GF14ε was slightly lower than GF14ω and this was also the case for the in silico prediction of secondary structure. In fact, the predicted GF14ω α-helical content was 64,86%, whereas that of GF14ε was 54,33%.

### Role of the C-terminal domain in the isoforms specificity towards the H^+^-ATPase

**Figure 3:**
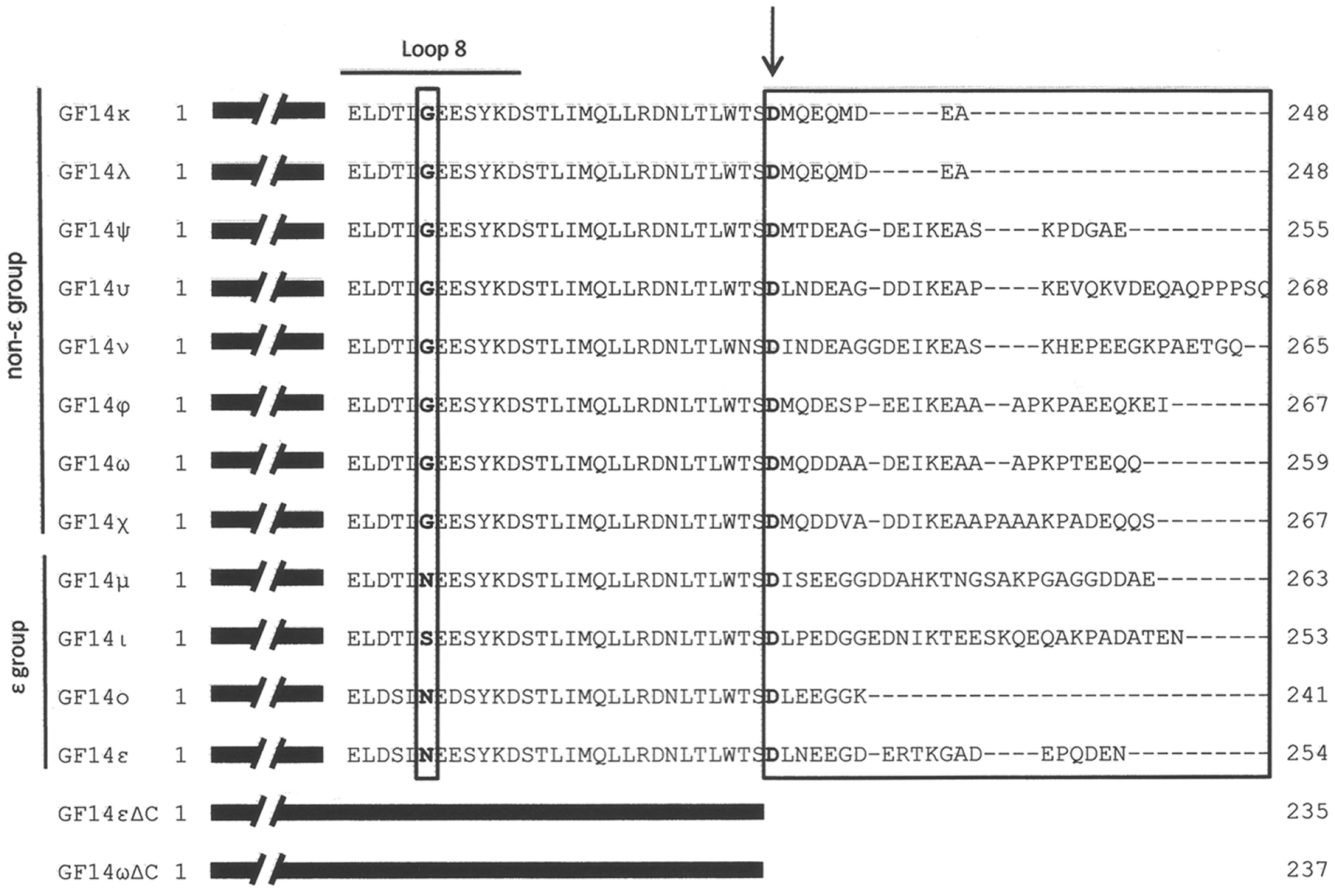
Amino acid sequence alignment of the C-terminal domains of *Arabidopsis* GF14 isoforms. The hypervariable C-terminal tails are boxed. The critical loop 8 residue, which is a conserved Gly in all non-ε isoforms, is in bold and boxed. The arrow marks the Asp residue that has been converted into a Stop codon in the GF14 deletion mutants. GF14εΔC, is 20 amino acids shorter than GF14ε; GF14ωΔC is 23 amino acids shorter than GF14ω.

**Figure 4:**
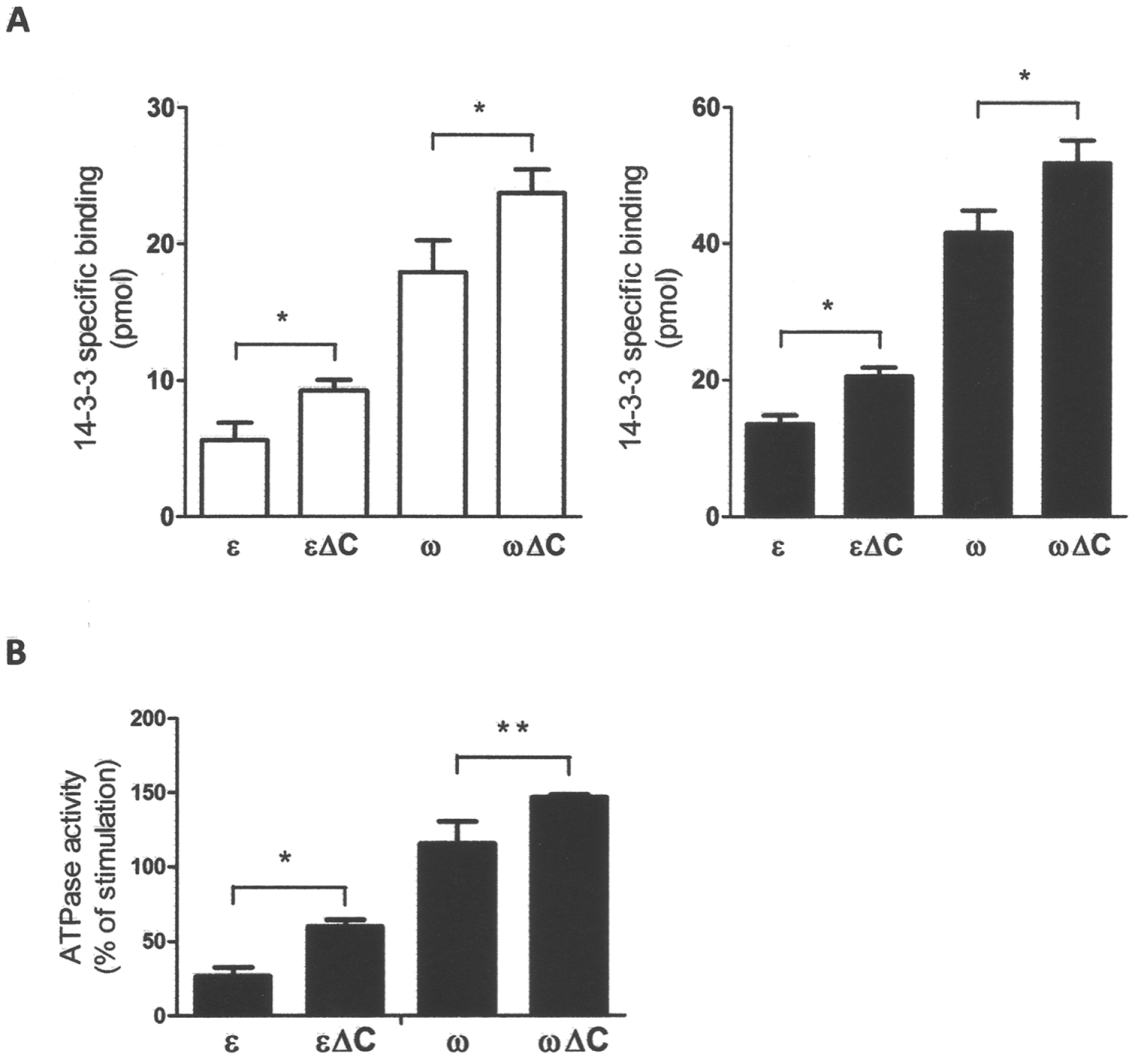
Effect of C-terminal deletion on the binding properties of GF14ε and GF14ω. A. Peptide binding assay: 0.05 nmol of bL15Vp biotinyl-peptide were immobilized onto streptavidin–agarose magnetic beads and incubated with 0.1 nmol wild type ^32^P-labeled-GF14ε and ^32^P-labeled-GF14ω or corresponding C-terminal deletion mutants in the presence or in the absence of 10 µM FC. Data are the means ± S.E. of three independent experiments. *, p<0,01. B. The stimulatory effect of GF14εΔC and GF14ωΔC on the phosphohydrolytic activity of AHA1 was compared to that of corresponding full-length proteins. Ten µg of ER membranes containing AHA1 H^+^-ATPase were incubated with 0.1 µM wild type and mutated GF14 proteins in the presence of 10 µM FC. Values are expressed as a percentage of stimulation of the basal H^+^-ATPase activity measured in the absence of 14-3-3 proteins. Data are the means ± S.E. of four independent experiments. *, p<0,01; **, p<0,05.

Previous reports have suggested a role for the divergent C-terminal domain of 14-3-3 isoforms in different ligand binding and discrimination [30], [32], [33]. To verify whether the different behaviour of GF14 isoforms in their binding ability and stimulation of H^+^-ATPase may be the consequence of their divergent C terminus, C-terminal deletion mutants were produced and used in interaction studies. GF14ω deleted of the last 23 amino acid residue (GF14ωΔC) and GF14ε deleted of the last 20 amino acid residues (GF14εΔC) (Figure 3) were expressed in *E. coli* as GST-fusion proteins to be compared to wild type isoforms in binding assay with the immobilized bL15Vp peptide, in the presence and in absence of 10 µM FC. Both GF14 isoforms deleted of their C-terminal tail showed an increased binding ability (Figure 4A), independently of FC. The consequence of this higher binding capability was, as expected, an increased stimulatory effect on the AHA1 phosphohydrolytic activity (Figure 4B). These results confirmed previous data on the autoinhibitory properties of the 14-3-3 C-terminal domain [30]–[32]. Interestingly, the effect of C-terminus removal on GF14 activity was higher in ε (60% in the absence of FC, 52% in the presence of FC) than in the ω isoform (31% in the absence of FC, 25% in the presence of FC), thus indicating that the C-terminal regions of the two isoforms exert a different autoinhibitory effect. The higher autoinhibition of GF14ε cannot be the unique responsible of the lower binding ability displayed by this isoform, since the binding ability of both deletion mutants, although increased compared to full-length proteins, was still different.

**Figure 5:**
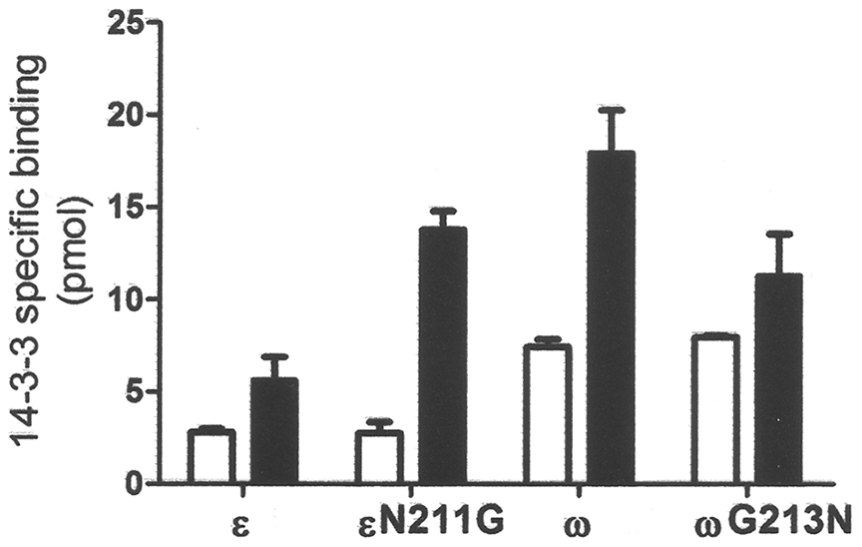
Role of Gly residue in loop 8 of non-ε isoforms in the interaction with H^+^-ATPase. 0.05–agarose magnetic beads and incubated with 0.1 nmol ^32^P-labeled wild type GF14ε and GF14ω isoforms and with the corresponding GF14εN211G and GF14ωG213N mutants in the presence or in the absence of 5 mM Mg^2+^. Data are the means ± S.E. of three independent experiments.

The association of 14-3-3 proteins with the H^+^-ATPase was Mg^2+^-dependent [40] and stimulated by spermine [34]. The ability of 14-3-3 proteins to bind cations appeared to depend on the presence of a putative EF hand-like domain, located in the loop 8 of the protein [35]. In this domain a central Gly residue is conserved exclusively in non-ε isoforms [36], while the ε isoforms, with the exception of GF14ι, are characterized by the presence of an Asn residue at the corresponding position, as shown in Figure 3. These residues were mutated in order to investigate their role in cation sensitivity of GF14 interaction with the target. GF14ωG213N and GF14εN211G mutants were expressed as GST-fusion proteins in *E. coli* and compared to corresponding wild type proteins for their ability to bind the bL15Vp peptide in the presence or in the absence of 5 mM Mg^2+^. As shown in Figure 5, the presence of Mg^2+^ affected the binding properties of both isoforms. As expected, Mg^2+^ addition increased the amount of GF14ε and GF14ω bound to bL15Vp but, notably, GF14εN211G mutant appeared to be even more sensitive to the divalent cation (Figure 5). In fact, while in the absence of Mg^2+^ the mutant isoform bound bL15Vp at a similar extent of wild type GF14ε, in the presence of Mg^2+^ the amount of protein bound to peptide was more than fivefold higher. Accordingly, substitution of the Gly213 in GF14ω to obtain the GF14ωG213N mutant reduced the effect of divalent cation on the binding property of the protein. The stimulatory effect of Mg^2+^ on wild type GF14ω was significantly higher than that observed on GF14ωG213N. These results strongly suggest that the Gly residue in the loop 8 of non-ε GF14 isoforms has a relevant role in their binding properties; this concurs with data reported on the isoform GF14 μ where substitution of Asn213 residue with Gly produced a mutant displaying a more flexible C-terminal region and a different target specificity compared to wild type [36].

### Isoform specificity of GF14 proteins towards other *Arabidopsis* targets

The different behaviour of ε and non-ε isoforms towards the H^+^-ATPase could be more generally shared by other 14-3-3 *Arabidopsis* targets. To investigate this possibility, a phosphopeptide array, obtained by using the SPOT-synthesis technology, was tested with different GF14 isoforms. The peptide array consisted of eight repeats of 21 tridecapeptides (reported in Table 2) containing known or putative 14-3-3 binding sites of *Arabidopsis* target proteins chosen from the literature data. Some peptides were designed based on 14-3-3 binding site identified in orthologous 14-3-3 clients in different plant species, others were designed on putative binding sites identified in the *Arabidopsis* target protein either on the basis of homology with 14-3-3 mode I or mode II binding motifs or because they were known to be *in vivo* phosphorylated. As peptides are immobilized by their C-termini, the mode III binding sites where the C-terminus is directly involved in the interaction were not considered.

**Table 2:** Peptides used in the peptide array binding assay with GF14 isoforms.

14-3-3 targets	14-3-3 binding peptides	References
NR	1 *PSLKKSVpSTPFMN	[51]
TPS5	2 *–MVSRpSYSNLL	[51]
TPS5	3 *KRFPRVApTVTGVL	[51]
F2KP	4 *RSLSASGpSFRNDS	[62]
F2KP	5 *PRLVKSLpSASSFL	[62]
SPS	6 **DHMPRIRpSEMQIW	*So) [56]
SPS	7 **DLLTRQIpSSPEVD	*So) [56]
GS1	8 **HNAAKIFpSHPDVA	*Bo) [57]
GS2	9 **GIDLRSKpSRTIEK	*Bo) [57]
GADPH	10 GAKKVVIpSAPSKD	[58]
Glu-tRNA synthetase	11 VIVRFDDpTNPAKE	[58]
BZR1	12 *PSLRISNpSCPVTP	[52]
TPK1	13 *RRLRRSRpSAPRGD	[53]
CPK1	14 DREIRTEpSKPET	[60]
ATP synthase β-subunit (plastid)	15 RKIQRFLpSQPFHV	[63]
Phot1	16 **KKPARRMpSENVVP	Vf) [64]
Phot2	17 *SKRRRSKpSQPLPT	[54]
ABF3	18 *QCLRRTLpTGPW–	[55]
CDC48	19 IFDKARQpSAPCVL	[60]
SERK1	20 CLRERPPpSQPPLD	[60]
KAPP	21 YKQRLPSpSSPHFS	[60]

For all 14-3-3 target proteins, the interaction with *Arabidopsis* GF14 proteins was demonstrated. *, peptides reproducing 14-3-3 binding site identified in *Arabidopsis* 14-3-3 clients. **, peptides gathered from an alignment of identified binding sequence of 14-3-3 clients derived from different plant species and the corresponding *Arabidopsis* targets. All the other peptides reproduced putative 14-3-3 binding site, present in the indicated *Arabidopsis* 14-3-3 target, and chosen based on their similarity with the 14-3-3 mode I or mode II binding motif.

NR, nitrate reductase; TPS5, trehalose phosphate synthase 5; F2KP, fructose-6-phosphate,2-kinase/fructose-2,6-bisphosphatase; SPS, sucrose-phosphate synthase; GS1, glutamine synthetase 1; GS2, glutamine synthetase 2; GADPH, glyceraldehydes-3-phosphate dehydrogenase; BZR1, brassinazole-resistant 1 protein; TPK1, two pore K^+^ channel 1; CPK1, calcium-dependent protein kinase 1; phot1, phototropin 1; phot2, phototropin 2; ABF3, ABA-responsive-element Binding Factor 3; SERK1, somatic embryogenesis receptor-like kinase 1; KAPP, kinase associated protein phosphatase.

So, *Spinacia oleracea*; Bo, *Brassica oleracea*; Vf, *Vicia faba*.

All peptides were phosphorylated on a single Ser or Thr residue, positioned in the center of the sequence. Binding assay was performed by incubating each array with a GST-GF14 isoform or GST alone, as a control. Peptide-bound proteins were revealed by an anti-GST antibody conjugated to peroxidase.

**Figure 6:**
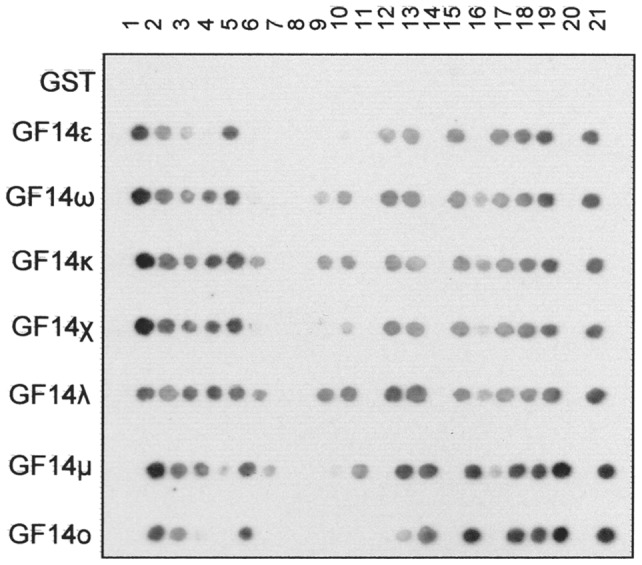
Peptide array binding assay with GF14 isoforms. Peptides (1 to 21, listed in Table 2) were synthesized by the SPOT-synthesis method and probed for binding to GF14 isoforms fused to GST. Each array was incubated with a different GST-GF14 isoform or GST alone, using 0.2 µM protein. Binding of GF14 isofoms was detected by anti-GST antibodies conjugated to peroxidase and a chemo-luminescence substrate.

As shown in Figure 6, GF14 isoforms differently bound to the peptides. As expected, GF14 isoforms bound to peptides reproducing the 14-3-3 binding site of nitrate reductase (NR: 1) [51], trehalose-phosphate synthase 5 (TPS5: 2, 3) [51], brassinazole-resistant 1 protein (BZR1: 12) [52], two pore K^+^ channel (TPK1: 13) [53], phototropin 2 (Phot2: 17) [54] and ABA-responsive-element Binding Factor 3 (ABF3: 18) [55], in agreement with the literature data identifying these sequences as 14-3-3 binding sites, thus confirming the validity of this approach for investigating the *in vitro* interaction of 14-3-3 proteins with their targets.

Some of the hypothetical 14-3-3 binding sites, although related to mode I or mode II binding motifs, did not interact in the assay: no binding was observed with the putative binding sequence of *Arabidopsis* sucrose-phosphate synthase (SPS: 7) and cytosolic glutamine synthetase (GS1: 8) – gathered from alignment with the binding site in the *Spinacia oleracea* [56] and *Brassica oleracea* [57] orthologs respectively –, the putative 14-3-3 binding site of *Arabidopsis* Glu-tRNA synthetase (11) [58], calcium-dependent protein kinase-1 (CPK-1: 14) [59] and embryogenesis receptor-like kinase 1 (SERK1: 20) [60]. Nevertheless this result was not totally unexpected, since these peptides contain residues in specific positions negatively affecting 14-3-3 binding, as Yaffe et al. [5] and, more recently, Panni et al. [61] demonstrated using degenerate peptide libraries. The Glu-tRNA synthetase peptide (11) has two negatively charged residues at −1 and −2 positions, with respect to the phosphorylated residue, as well as an Asn residue at +1 position, all features known to reduce the affinity with 14-3-3 proteins. Similarly, the CPK-1 peptide (14) contains a Glu residue at −1 and a Lys residue at +1 position, while the SERK1 peptide (20) contains a Pro residue at −1 position, which markedly hamper 14-3-3 binding.

Interaction was instead detected with the putative binding sequences of fructose-6-phosphate,2-kinase/fructose-2,6-bisphosphatase (F2KP: 4 and 5) [62], glyceraldehydes-3-phosphate dehydrogenase (GAPDH: 10) [58], ATP synthase β-subunit (15) [63], phototropin 1 (Phot1: 16) [64], AAA-ATPase CDC48 (19), PP2C kinase-associated protein phosphatase (KAPP: 21) [60] and, even to a very low extent, the plastidic glutamine synthetase (GS2: 9) [58].

Interestingly both the F2KP putative binding sites interacted with GF14 proteins. These two sequences have been demonstrated to be *in vivo* phosphorylated, but the direct evidence of their involvement in 14-3-3 binding has thus far been lacking and only the sequence surrounding the Ser303, corresponding to peptide 5, has been proposed as 14-3-3 binding site for its similarity with the 14-3-3 mode I binding motif [62].

In addition the ATP synthase β-subunit sequence has been supposed to be a 14-3-3 binding site based on its homology with the mode I motif [63] and here is shown to be directly involved in 14-3-3 binding.

**Figure 7:**
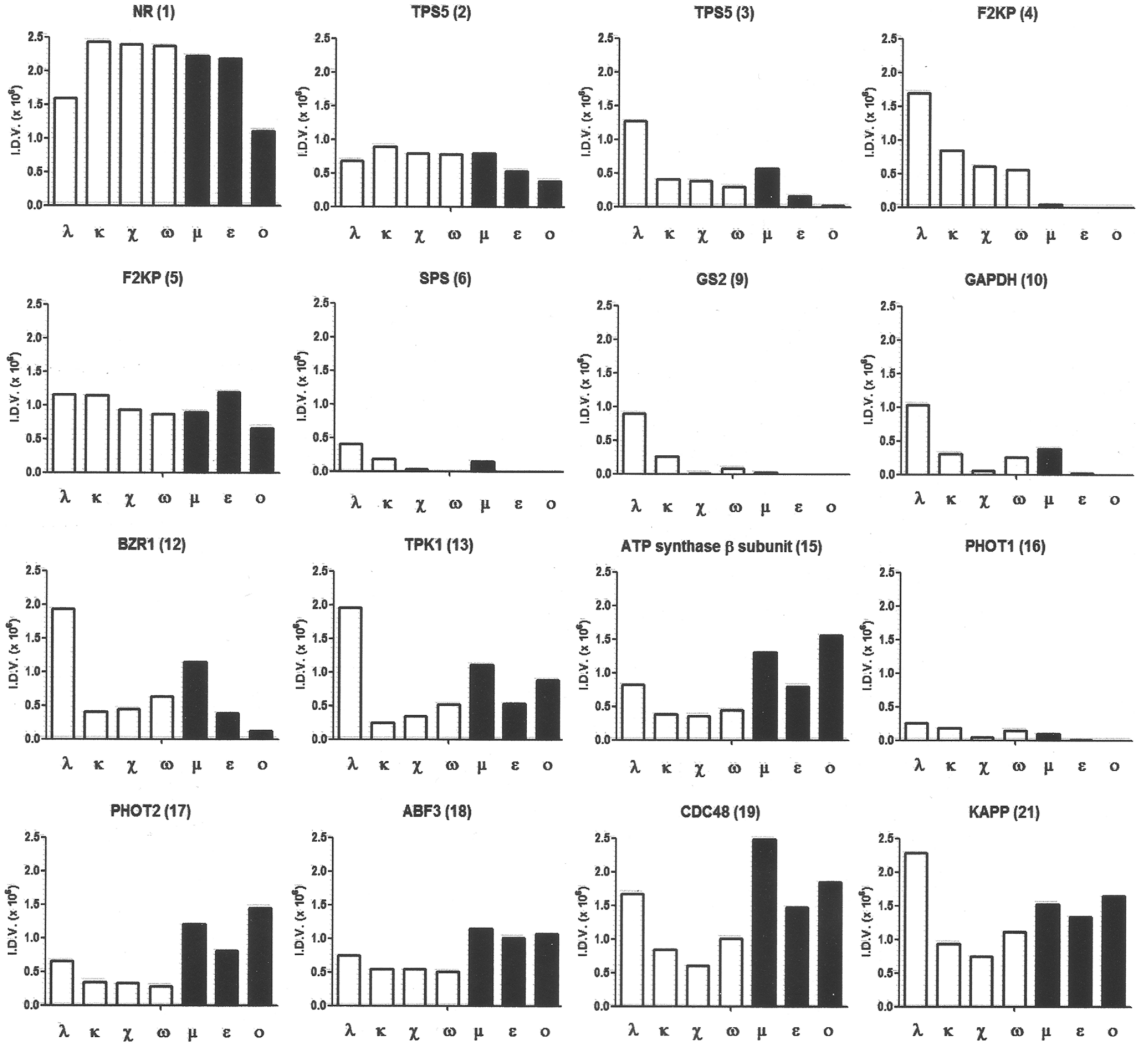
Data analysis of the peptide array binding assay. Densitometric analysis of spots derived from the experiment in Figure 6. Data are expressed as Integrated Densitometric Value (the product of the area and mean gray value).

Notably, in some cases GF14 isoforms differently interacted with the same peptide. Densitometric analysis of the positive spots allowed for better evaluating the differences in the GF14 interactions (Figure 7). Interestingly, with some peptides GF14 isoforms belonging to the same phylogenic ε and non-ε group displayed similar interaction capability, suggesting the existence of a “group specificity”, similarly to that observed with the AHA1 H^+^-ATPase. In particular, with the ATP synthase β-subunit (15), phot2 (17) and ABF3 (18) peptides the interaction of all GF14 ε isoforms was higher respect to that of all non-ε isoforms, whereas F2KP (4) and GS2 (9) peptides interacted exclusively with isoforms belonging to the non-ε group.

It is worth noting that GF14λ, among the non-ε isoforms, was more active in most of the interactions tested. Moreover, with BZR1 (14), TPK1 (15) and KAPP (21) peptides GF14λ displayed a different behaviour compared to the other non-ε members, in fact its interaction was the highest while the other non-ε isoforms are less active than ε isoforms.

## Discussion

The existence of multiple isoforms of 14-3-3 proteins has raised the question on their functional role. Besides the different spatial expression pattern in various tissues and organs, during the plant development or in response to external stimuli, some isoform specificity in target recognition has been reported [65].

In the present work, the plasma membrane H^+^-ATPase was chosen to investigate the functional role of *Arabidopsis* GF14 isoforms, being one of the best characterized 14-3-3 client in plants. We demonstrate that GF14 isoforms bind to the H^+^-ATPase and stimulate its activity in an isoform-specific manner; in particular, both in binding and stimulation of the enzyme, all tested GF14 non-ε isoforms resulted more active than GF14 ε group. Previous data showed some different results on isoform specificity towards the H^+^-ATPase [23], [66]. A rationale for this may be that those experiments had been performed with peptides reproducing the AHA2 (*Arabidopsis* H ^+^-ATPase isoform 2) C-terminus, while in the present work a peptide reproducing the maize MHA2 C-terminus and the AHA1 enzyme were used in interaction and enzymatic activation studies, respectively. Differences in target sites, even slight, as those present in the H^+^-ATPase C-termini, could affect binding selectivity.

The observation that isoforms that are more active in H^+^-ATPase binding and activation belong to the same phylogenetic group allows us to propose a “group specificity” towards the H^+^-ATPase. Results from the peptide array interaction studies are in agreement with the different behaviour of GF14 isoforms depending on the target peptide. Interestingly, also in these experiments isoforms belonging to the same group shared similar binding affinities to some peptides reproducing binding sites of several targets and supporting the hypothesis of a “group specificity” in several interactions. This appears particularly evident for one of the two 14-3-3 binding sites (peptide 4) in F2KP that interacts exclusively with non-ε isoforms. It is interesting to note that its sequence is not a 14-3-3 mode I motif. The presence in F2KP of two 14-3-3 binding sites exhibiting a different GF14-specificity raises questions regarding their functional role. The regulation of F2KP activity appears in fact of great interest for the pivotal role played by fructose-2,6-bisphosphate in the control of plant sugar metabolism.

Additional peptides, such as the ATP synthase β-subunit (peptide 15) and Phot2 (peptide 17) show a slight preferential interaction with isoforms belonging to the ε group. The interaction of ATP synthase β-subunit peptide with ε group isoforms is in accordance with previous studies demonstrating that GF14ε and μ isoforms, but not GF14ω, can be localized within the plastidic stroma, where they also participate in the regulation of starch synthase activity [67], [68].

A similar preferential interaction with isoforms belonging to the ε group is evident also for the ABF3 peptide (18). Nonetheless, studies of ABFs interaction with 14-3-3 proteins of *Thellungiella salsuginea*, a close relative of *Arabidopsis*, performed by yeast two-hybrid system show a specific interaction with non-ε isoforms. Since in these studies ABF2 and ABF4, but not ABF3, were used [27] it is possible that different target isoforms could display different specificity towards 14-3-3 isoforms.

Besides the “group specificity”, it is also of interest that isoforms belonging to the same phylogenetic group displayed a different behaviour in the interaction with some peptides. In particular, GF14λ was often the most active among the non-ε isoforms and, in several cases, such as TPK1 (13) and BZR1 (14) peptides, the difference compared to the other isoforms was considerable. Accordingly, Latz et al. [53] showed, by SPR analysis, that TPK1 interaction had a K_D_ that was more than 10 fold lower for GF14λ than GF14χ, another non-ε isoform. Interestingly, although GF14κ is strongly related (93% identical in the primary structure) to GF14λ, it does not exhibit the same high binding affinity.

The specific biochemical binding properties of GF14λ may explain the peculiar behaviour of this isoform *in vivo*: GF14λ, but not GF14κ, seems to be required for stomatal opening mediated by Phot2 [54].

At variance with results on peptides used in the array, GF14λ does not exhibit a higher activity compared to the other GF14 non-ε isoforms in the interaction with the H^+^-ATPase and in its stimulation (Figure 1).

A more detailed study on the kinetic and thermodynamic properties of the interaction between the ε and non-ε isoforms and the H^+^-ATPase was performed by SPR analysis. The results concerning the interaction between the peptide reproducing the 14-3-3 binding site of the H^+^-ATPase and GF14ε or GF14ω, as representative of ε and non-ε isoforms, respectively, demonstrate that GF14ω has a higher affinity and that this was due to a slower dissociation rate of the complex, as can be deduced by comparing the kinetic parameters determined for both isoforms (Table 1). The different affinity of GF14 isoforms was particularly evident when FC was added, the K_D_ value determined for GF14ω (K_D_  = 4.1 nM) was about 25 fold lower than that of GF14ε and similar to that reported in literature for a maize 14-3-3 isoform (K_D_  = 7 nM) [46]. As known, FC stabilizes 14-3-3/H^+^-ATPase complex, slowing down the dissociation phase, as demonstrated by the decrease in k_off_ values of two order of magnitude for GF14ω and one order of magnitude for GF14ε. Notably, FC seems to have a negative effect on the association rate, being that the k_on_ values for both isoforms were reduced, even slightly, after toxin addition. This finding indicates that FC slows down the 14-3-3/H^+^-ATPase complex formation, but strongly reduces its dissociation.

During the past number of years, the molecular mechanism underlying the isoform binding specificity has been investigated but a full clarification has not yet been obtained. Structural data reported for mammalian and plant 14-3-3 proteins indicate that residues directly involved in the ligand binding are highly conserved among isoforms [11], [12], [17]. Obsil and co-workers [69] observed that in the crystal structure of the 14-3-3ζ:Serotonin N-acetiltransferase complex, of the 37 residues of 14-3-3 contacting the target, 34 are identical in the mammalian 14-3-3 isoforms and the other four conservatively substituted. Despite that, *in vivo* serotonin N-acetiltransferase were preferentially found associated with 14-3-3ε and 14-3-3ζ isoforms, suggesting that structural characteristics outside the binding groove may be responsible for isoform binding specificity.

Results shown in the present work indicate that both the C-terminal domain and a specific Gly residue, within the loop 8 of 14-3-3s, play a role in the GF14 isoform specificity.

The primary structure of 14-3-3 isoforms show the high divergence of C-terminal domains which has already been proposed to be involved in ligand binding and discrimination. Our results with GF14ε and GF14ω deletion mutants demonstrate that the C-terminal region plays an autoinhibitory role, which accords with literature data obtained with other 14-3-3 isoforms [30]–[32]. It is worth noting that the effect of the C-terminal removal is higher on GF14ε compared to GF14ω (Figure 4), thus suggesting that the C-terminal region can have a role in the isoform specificity because of a different extent of autoinhibition. Otherwise, it can be directly involved in ligand interaction as demonstrated by a peptide reproducing the C-terminal region of a maize 14-3-3 affecting H^+^-ATPase activity [32] and by the observation that the GF14ω C-terminal tail is required for nitrate reductase inhibition [33].

The position of the C-terminal region, and consequently the groove accessibility to the target, can be altered by binding of divalent cations at the loop 8 of 14-3-3. A Gly residue, within this loop, is necessary for the correct folding of the EF hand-like domain involved in cation binding; interestingly this residue is specifically conserved in non-ε isoforms. Substitution of this Gly in GF14ω isoform with an Asn residue, present at the same position in GF14ε, reduces GF14ω binding to the H^+^-ATPase (Figure 5). This effect is due to a lower Mg^2+^ sensitivity, since it can be detected only in its presence. Accordingly, replacement of Asn for Gly within the loop 8 of GF14ε increases its Mg^2+^-dependent interaction with the H^+^-ATPase. This result well confirms data previously obtained by Sehnke et al. [36] with the GF14 μ isoform and its target nitrate reductase, suggesting that the Gly in the loop 8 might affect the C-terminal flexibility of 14-3-3 proteins.

## Conclusions

This paper demonstrate that *Arabidopsis* 14-3-3 isoforms, classified in ε and non-ε groups, display a “group specificity” in the target recognition. This is observed in the FC-mediated activation of the H^+^-ATPase, one of the best-characterized 14-3-3 clients in plants, and confirmed by probing several targets by SPOT peptide array method. The results reported show that the isoform binding specificity is dependent on structural characteristics outside the binding groove, in particular the C-terminal domain and a specific Gly residue, within loop 8 of 14-3-3s. The C-terminal region can play a role in the isoform-specificity because of a different extent of autoinhibition. Moreover, the different C-terminal flexibility and the extent of α-helix content appears to play an important role in their differential activity.
